# Evaluation of Bosniak Type IIF and III Renal Cysts with Contrast-enhanced Ultrasound

**DOI:** 10.5334/jbr-btr.843

**Published:** 2016-02-01

**Authors:** Hakan Öztürk

**Affiliations:** 1 Sifa University, TR

**Keywords:** Bosniak, classification, Contrast-enhanced Ultrasound, diagnosis

## Letter

**Figure 1:**
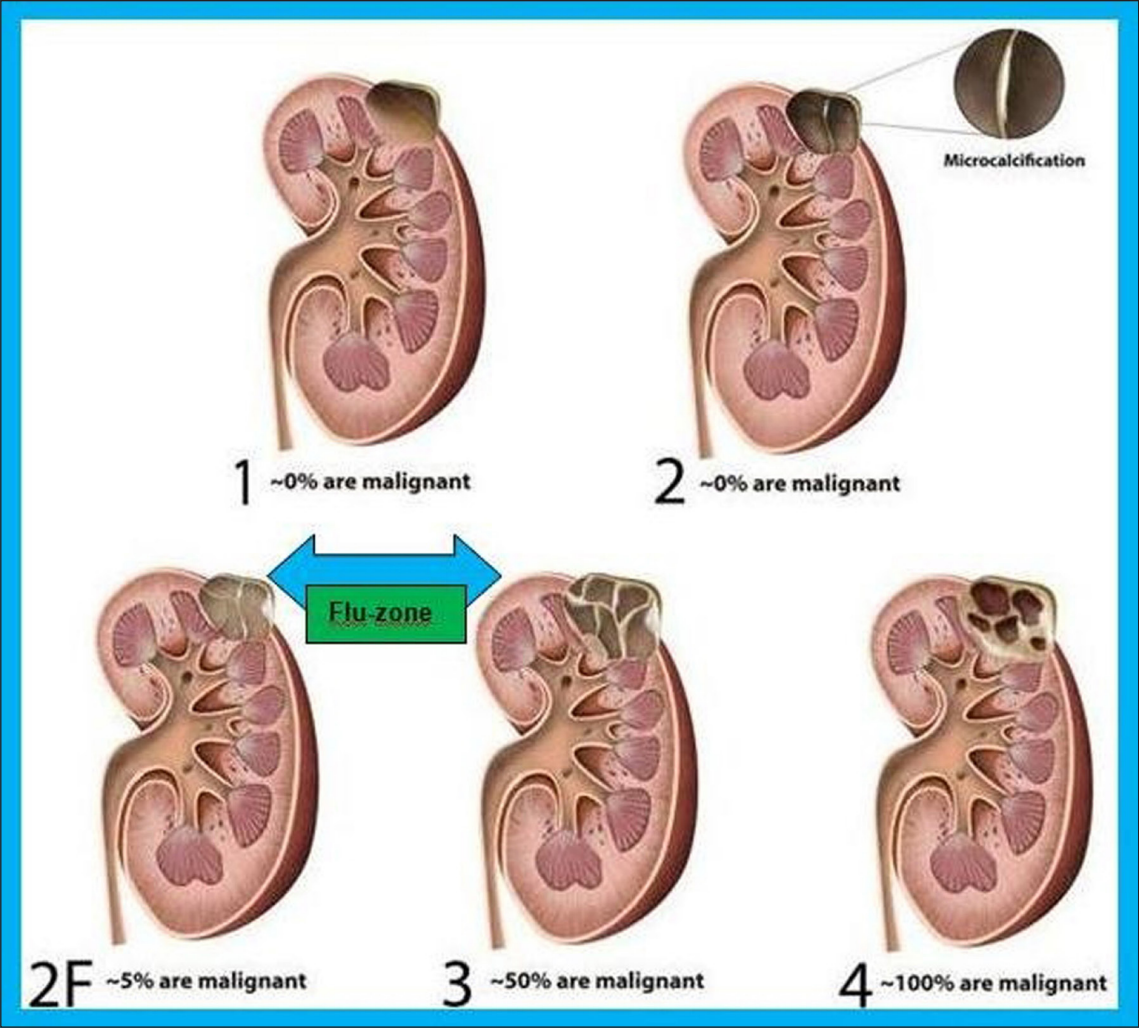
Schematic representation of Bosniak type I–IV cysts.

An increase in the segmentation visualization techniques resulted in a parallel, more frequent determination of complex renal cysts. Renal cysts were classified by Bosniak, and this classification was modified in 2003, in order to attempt to narrow the large interval between type II and type III by the definition of IIF category [1]. However, none of the recent diagnostic modalities could clarify the grey-zone between type IIF and type III lesions (Figure 1). The main disadvantage of CT in this state is the determination of false contrast-enhancement in about 22% of the cysts. This situation is a special concern when the intraparenchymal cysts are smaller than 1 cm [2]. There are technical disadvantages for magnetic resonance imaging (MRI) and diffusion weighted imaging (DWMRI). For example, cysts with dense proteinaceous content cannot be differentiated from malignant lesions by the DWMRI technique [2]. Another of the problems using CT and MRI is the possibility to fail to notice the contrast involvement due to the limited sampling and timing of contrast material administration. Contrast-enhanced ultrasound (CEUS), which became frequently used in recent years, provides potential solutions to this problem.

CEUS require the bolus injection of microbubbles made up of protein, lipid, or polymer shell and sulfur hexafluoride gas, and then the real-time determination of the echogenicity changes caused by these microbubbles. When exposed to US waves, the dimensions of these microbubbles enlarge twice; this oscillation causes a reflection in the US transducer. Specific software allows elimination of the background tissue, and easy visualization of the minimum quantity of US contrast trapping [3]. These agents are neither nephrotoxic nor hepatotoxic [3]. The half-life of these agents is approximately five minutes, which gives way to multiple injections during one session, and thus verification of the suspected lesion with high accuracy. An additional evaluation of patients with Bosniak IIF and III lesions, by contrast-enhanced US, may help to classify the lesions with a high degree of precision—decreasing the need for follow-up and helping lesion reclassification.

According to the current scientific data related with CEUS; it may be as valuable as contrast-enhanced CT, and even in determining contrast trapped by thin septa in complex cysts, and in determining the tumor vascularity. Quaia et al. reported that diagnostic accuracy of CEUS is 89% and that of CT is 69% [2]. Park et al. compared CEUS and CT of complex cystic renal masses, and determined that diagnostic accuracy of CEUS and CT for malignant renal tumors were 90% and 74%, respectively [2]. In a study by Barr et al. including 1018 lesions, 306 pathologically confirmed lesions were evaluated by CEUS, yielding a sensitivity of 100%; specificity was 95.2%, positive predictive value was 94.6%, and negative predictive value was 100% [4]. Barr et al. indicated that Bosniak III lesions that do not show CEUS contrast trapping can reasonably undergo follow-up. Additionally, it was reported that every lesion presenting contrast trapping on CEUS should be considered malignant until proven otherwise, and undergo biopsy and/or surgical removal, independently of its Bosniak score [4].

CEUS may help reclassifying of lesions that appear to be biopsied or surgically resected, to lesions that must be followed-up. The high diagnostic values obtained by CEUS in evaluating malignancy in Bosniak type IIF and III cystic renal masses, may decrease the need for CT or MRI. Although contrast-enhanced CT remains the standard of reference for classification, CEUS holds promises for a better determination of the lesion characteristics, such as wall and septum thickness. It is considered that CEUS findings will be added to the future classification parameters, especially for the differential diagnosis of type IIF and type III cysts.

## Competing Interests

The author declares that they have no competing interests.

## Abbreviations

Ultrasound (US)

Contrast-enhanced Ultrasound (CEUS)

Computed Tomography (CT)

Magnetic resonance imaging (MRI)

Diffusion weighted magnetic resonance imaging (DWMRI)

## References

[1] Israel GM, Bosniak MA (2003). Follow-up CT of moderately complex cystic lesions of the kidney (Bosniak category IIF). AJR Am J Roentgenol.

[2] Ellimoottil C, Greco KA, Hart S, Patel T, Sheikh MM, Turk TM (2014). New modalities for evaluation and surveillance of complex renal cysts. J Urol.

[3] Cokkinos DD, Antypa EG, Skilakaki M, Kriketou D, Tavernaraki E, Piperopoulos PN (2013). Contrast enhanced ultrasound of the kidneys: what is it capable of?. Biomed Res Int.

[4] Barr RG, Peterson C, Hindi A (2014). Evaluation of indeterminate renal masses with contrast-enhanced US: a diagnostic performance study. Radiology.

